# Electrochemical Glucose Sensors—Developments Using Electrostatic Assembly and Carbon Nanotubes for Biosensor Construction

**DOI:** 10.3390/s100908248

**Published:** 2010-09-02

**Authors:** Alice Harper, Mark R. Anderson

**Affiliations:** 1 Department of Chemistry, Berry College, 2277 Martha Berry Highway, P.O. Box 5016, Mt. Berry, GA 20149, USA; E-Mail: asuroviec@berry.edu (A.H.); 2 Department of Chemistry, University of Colorado Denver, 2190 E. Iliff Ave., Denver, CO 80217-3364, USA

**Keywords:** glucose oxidase, electrochemical sensors, electrostatic assembly, carbon nanotubes

## Abstract

In 1962, Clark and Lyons proposed incorporating the enzyme glucose oxidase in the construction of an electrochemical sensor for glucose in blood plasma. In their application, Clark and Lyons describe an electrode in which a membrane permeable to glucose traps a small volume of solution containing the enzyme adjacent to a pH electrode, and the presence of glucose is detected by the change in the electrode potential that occurs when glucose reacts with the enzyme in this volume of solution. Although described nearly 50 years ago, this seminal development provides the general structure for constructing electrochemical glucose sensors that is still used today. Despite the maturity of the field, new developments that explore solutions to the fundamental limitations of electrochemical glucose sensors continue to emerge. Here we discuss two developments of the last 15 years; confining the enzyme and a redox mediator to a very thin molecular films at electrode surfaces by electrostatic assembly, and the use of electrodes modified by carbon nanotubes (CNTs) to leverage the electrocatalytic effect of the CNTs to reduce the oxidation overpotential of the electrode reaction or for the direct electron transport to the enzyme.

## 1. Introduction

As health-care shifts increasingly away from the clinical setting, the availability of diagnostic tools that can accurately determine medical conditions, are easy to operate and evaluate the response of, and can be used by an untrained individual becomes increasingly important. Biosensors based on electrochemical signal transduction meet many of these requirements and represent an area of active research.

Electrochemical biosensing has its origins in a report in 1962 by Clark and Lyons that describes a potentiometric measurement coupled with the enzyme glucose oxidase to determine glucose in blood plasma [1]. This report also introduced the concept of coupling an enzyme with the signal transduction in the creation of a sensor [1]. The success of enzyme-coupled electrochemical sensors is due in part to the excellent selectivity that enzymes have for analytes of biomedical interest, as well as the simplicity and sensitivity of the electrochemical measurement [1]. Since the report by Clark and Lyons, research on enzyme based glucose sensors has been extensive. One reason for this research activity is the number of Americans with diabetes. According to the American Diabetes Association, as of 2007 (the last year that complete diabetes data is available), 23.6 million children and adults in the United States, 7.8% of the population, have diabetes [2].

Coupling an enzyme to the signal transduction adds the specificity of the enzyme for a particular substrate to the measurement. In general, a biosensor contains a molecular–recognition element directly interfaced to a signal transducer which, together produce a response proportional to the concentration of analyte [3,4]. In an electrochemical biosensor, an electrode serves as the signal transducer, where the measurable response is either an electrical current due to a redox reaction (with an amperometric sensor), or to the change in electrode potential (with a potentiometric sensor). The choice of molecular–recognition element depends on the analyte, and may range from redox proteins (cytochrome c) to enzymes (glucose oxidase) to DNA [4–7].

Electrochemical sensors are attractive due to their low-cost, relatively fast time-response, their operational simplicity, and the robust nature of electrochemical measurements [8]. There are, however, disadvantages with electrochemical sensors, particularly when coupled to an enzyme reaction. The most significant is the inefficient electron transfer between the molecular-recognition element, e.g., the enzyme, and the electrode surface. This slow electron-transfer efficiency is due to both the location of the redox active site deep within the enzyme and the inability of the enzyme to orient itself favorably with respect to the electrode surface for fast electron transfer [9]. Glucose oxidase (GluOx) is an example of an enzyme that is generally unable to be directly oxidized by an electrode that has been used frequently in construction of electrochemical biosensors. This review will provide a general overview of electrochemical glucose sensors, discuss experimental methods that have been implemented to overcome the limitations associated with coupling an enzyme reaction with electrochemical signal transduction, and specifically focus on developments for using electrostatic assembly and carbon nanotubes in the constructrion and evaluation of new glucose sensor assemblies.

Coupling the enzyme glucose oxidase (GluOx) with signal transduction has become a benchmark system in the development of biosensors [10–22]. GluOx has several favorable attributes that contribute to its common usage, including high turnover rate, excellent selectivity, good thermal and pH stability, and low cost [23]. The enzyme is comprised of two identical protein subunits and one flavin adenine dinucleotide (FAD) coenzyme molecule which is found in the active site. The FAD coenzyme molecule is tightly bound, yet not covalently attached to the enzyme [23,24]. Many other oxidase-type enzymes also incorporate a FAD coenzyme in the active site, making GluOx an excellent system for benchmarking new sensor designs.

FAD works efficiently as a cofactor because of its highly reversible electrochemistry. FAD can be reduced in a two electron, two proton process to form FADH_2_. During the enzymatic reaction between GluOx and glucose, the FAD is reduced by the glucose producing FADH_2_ and glucose is oxidized to glucono-d-lactone [25–28]. The FADH_2_ can then be oxidized by dissolved O_2_ producing H_2_O_2_, and returning the enzyme to its initial state containing FAD [27,28]. The Clark-Lyons demonstration of a sensor based on the reaction between glucose and glucose oxidase uses this reaction with potentiometric electrochemistry for the signal transduction [1]. Although reported nearly 50 years ago, this basic sensing scheme remains relevant today.

The crystal structure of GluOx is well-known and shows that the FAD sits in the funnel-shaped active site, with a 10 Å × 10 Å opening that tapers to an opening only a few Å across [24,29]. The size and shape of the active site enhances the specificity of GluOx for glucose. Unfortunately, due to the orientation of the FAD in the cleft, direct FAD oxidation by an electrode is kinetically unfavorable [30,31]. Calculations conducted by Heller show that electrons can be transported across distances as large as 20 Å; however, they also show that the distance between the FAD and the electrode surface is approximately 25 Å [9,32]. Marcus *et al.* show that the oxidation of an enzyme at an electrode surface suffers from low efficiency based on the limited distance that an electron can tunnel [9,32]. Despite the heterogeneous kinetic limitations, FAD oxidation is a thermodynamically favorable reaction, suggesting that other methods can be used to improve the efficiency of the enzyme reaction coupled to electrochemical measurements

Several general approaches to circumventing this kinetic limitation have been demonstrated. The enzyme can be confined to the electrode surface, and the action of the enzyme diagnosed indirectly by monitoring the formation of hydrogen peroxide from the enzyme-substrate reaction in the presence of O_2_ [33–35]. This method is an example of indirect glucose detection because the signal transduction is based on the oxidation of the H_2_O_2_ that is produced by the enzyme reaction in close proximity to the electrode surface, and not by directly monitoring the glucose. The stoichiometric relationship between glucose and H_2_O_2_ allows quantitative determination of the glucose from the current associated with the H_2_O_2_ oxidation. Unfortunately, this protocol for glucose detection suffers from the relatively high positive overpotential of H_2_O_2_ seen with most electrode materials, especially with carbon electrodes [23,36,37]. Due to the high overpotential of H_2_O_2_ oxidation, species such as ascorbic acid and acetaminophen, which are common in biological samples, may interfere with the electrochemical detection of the H_2_O_2_ [28]. The possibility of this interference encourages research into mechanisms for lowering the oxidation overpotential for H_2_O_2_ to introduce this separation.

Alternatively, an electron-transfer mediator which has a lower oxidation potential can be used to bridge the electrical communication between glucose oxidase and the glucose. When using a redox mediator, the measurement is conducted in an oxygen free solution to eliminate competition between the mediator and O_2_ for reoxidizing the FADH_2_. An electron-transfer mediator is a molecule that has fast electron transfer kinetics and that has an oxidation potential positive of the thermodynamic oxidation potential of the kinetically hindered reaction [3,28,38]. An important factor for making this thermodynamic process spontaneous is the difference in the oxidation potentials between the mediator and the FAD. The larger the gradient, the more favorable the homogeneous reduction-oxidation reaction between the mediator and the FADH_2_ will be. Under these conditions, the electrode oxidizes the mediator; then the oxidized form of the mediator is reduced by the FADH_2_. The net result is that the FADH_2_ is oxidized at a faster rate than is possible by heterogeneous electron transfer in the absence of the mediator.

A suitable mediator for the GluOx enzyme must have a redox potential more positive than the redox potential of the FAD [25,26]. The oxidation potential of the FAD center in the GluOx active site is −0.447 V or −0.337 V *vs.* Ag/AgCl, depending on the preparation [30,31]. The mediator must be able to rapidly cycle between its oxidized and reduced states in the process of shuttling charge between the oxidase and the electrode surface. The electron transfer from the enzyme to the mediator must also occur rapidly to overcome the kinetic barrier effectively [32]. The heterogeneous electron transfer kinetics of the mediator can potentially limit the rate of the reoxidation of the enzyme, so this step must also be kinetically favorable to keep the kinetic barrier low. Finally, the mediator must be stable in both of its oxidation states indefinitely for the mediation to continue [25,26]. Ferrocene and its derivatives are examples of mediators that are frequently used when constructing glucose electrochemical sensors since they have a wide range of redox potentials, are easy to derivatize, have fast electron transfer kinetics, and are stable in both the oxidized and reduced forms [25–27,31,39–44]. Examples of other redox mediators used with glucose oxidase based electrochemical sensors are given in Table 1.

**Figure 1.**
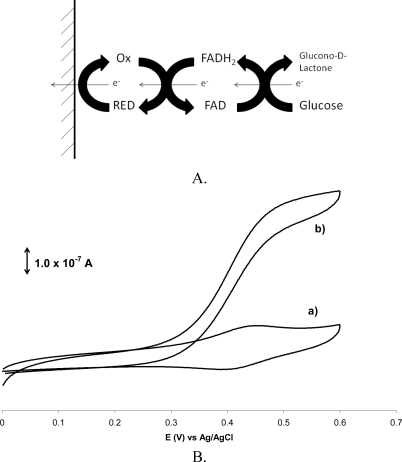
A. Schematic diagram of the redox mediation scheme. Glucose in the presence of Glucose oxidase is oxidized to Gluconolactone and the redox active FAD site in the enzyme is reduced to FADH_2_. The FADH_2_ can be oxidized back to FAD by reducing the redox mediator. The reduced form of the redox mediator is returned to the oxidized form by the heterogeneous electrode reaction. The current measured by the electrode reaction, therefore, is directly related to the amount of glucose that is oxidized by the enzyme reaction. B. (a) current response of the redox mediator in the absence of glucose, and (b) Steady state current response from the redox mediator in the presence of glucose [27]. (Alice C. Harper and Mark R. Anderson, “Electrostatic Assembly of a Redox Catalysis System for Detection of Glutamate”, Electroanalysis, 2006. Copyright Wiley-VCH Verlag GmbH & Co. KgaA. Reproduced with permission).

Constructing an electrochemical biosensor that takes advantage of a redox mediator requires only catalytic amounts of the mediator and the enzyme. Due to the electron shuttling, when glucose is added to the solution, the cyclic voltammogram of the mediator has a steady state response (Figure 1) [47]. This response by the electrode suggests that the rate of the reaction is limited only by the rate of the glucose oxidation by GluOx. This mediation scheme can be, in theory, used to facilitate many different enzyme reactions, provided that they contain a FAD center. The mediator is often dissolved in the electrolyte solutions to facilitate its mass transport between the electrode and the enzyme active site. Use of a soluble mediator, however, renders this sensor construction impossible for *in vivo* use.

Ferrocene-derivative mediators and GluOx can be incorporated into various types of matrices that are attached to an electrode surface so that the enzyme and mediator system are confined to a small volume adjacent to the electrode surface and cannot freely diffuse throughout the solution. The advantage of having both the mediator and the enzyme confined to the surface is that a higher degree of organization can be achieved, confining the system to a small dimension restricts the electron transfer distance between the mediator and the enzyme increasing the efficiency of the electron transfer mediation, and confining the mediator system to the electrode surface allows the possibility of the electrode being used *in vivo* [3,48]. The utility of systems with the mediator and enzyme bound into thin films have been illustrated by applications with *in vivo* measurements and microdialysis sampling [49–51]. Several ways to confine the enzyme and mediator system to the interface are found in the literature [19,51–58]. These include creating hydrogels or electropolymerization in the presence of the enzyme and the mediator to trap the enzyme and mediator within small volumes at the electrode surface, and the layer-by-layer deposition of polyelectrolytes.

Hydrogels are water insoluble cross-linked polymer films that are highly water absorbent. During the polymerization process, the resulting pores within the hydrogel encapsulate the enzyme or any other materials present in the solution [57–59]. By encapsulating the enzyme rather than covalently attaching it to the electrode surface, the enzyme can maintain its native conformation and properties. Often, a redox polymer that can serve as a redox mediator for the enzyme is also incorporated into the hydrogel matrix during the preparation [60–62]. Hydrogels are easily formed by standard polymerization methods [54,63–67]. Recently, hydrogels have also incorporated modified carbon nanotubes instead of a redox polymer to take advantage of the carbon nanotube’s electrocatalytic properties [60–62,68–70].

Electropolymerization is another method for trapping the enzyme at the electrode’s surface. This polymerization can take place either by a free radical reaction or by a condensation reaction depending on the monomers involved [11,51,71]. If the enzyme is present in the aqueous solution during the polymerization reaction, the enzyme can become encapsulated within the matrix at the electrode surface [71]. Polymer films incorporating enzymes have been made using several different monomers; however, systems making use of polyaniline and polypyrrole are most commonly found [11,71]. To incorporate GluOx in an electropolymerized film of polypyrrole, both pyrrole and GluOx are added to the electrolyte solution, and a small positive potential is applied to the electrode to polymerize the pyrrole [11,72–74]. The magnitude and duration of the applied potential dictates the film thickness and the amount of encapsulated GluOx. The resultant films are commonly characterized using cyclic voltammetry. Electropolymerization also has the ability to produce a film with more than one enzyme in a layer of polymer [25].

Occasionally the mediator is covalently coupled to the enzyme. This method attaches the mediator chain to the outer surface of the enzyme through an amide linkage [75–78]. These modified enzymes can be used in solution or integrated into a polymer matrix that is deposited onto the electrode surface. In either case, the modified enzyme must closely approach electrode surface to regenerate the mediator, often leading to low electron transfer rate between the electrode and the mediator when the mediator is covalently coupled to the enzyme [75–78].

All of these methods that confine the mediator and enzyme to the surface of the electrode are well developed and each has distinct advantages [3,79]. These enzyme-electrode attachment methods typically rely on encapsulating the enzyme in the pores of the polymer matrix. The ability of the enzyme to be oxidized by the mediator-containing polymer is determined by the enzyme being within the electron transport distance of the mediator. This can be addressed by covalently attaching the mediator to the enzyme; however, this restricts the movement of the mediator. It has been shown that the electron transfer in these kinds of biosensors can be slow due to concentration of the mediator in the matrix [3]. To circumvent the complications of these methods, a simpler technique utilizing electrostatics to construct an enzyme-mediator assembly composed of alternating layers of mediator containing polymer and the enzyme have been demonstrated.

Despite the successes of these preparations, innovations in glucose sensor construction and performance continue to appear. The following will focus on two areas of research that have seen new applications in sensor development during the last 10–15 years. Electrostatic assembly is a process in which polyelectrolytes can be easily deposited onto an electrode interface to form very thin molecular assemblies. As these assemblies are so thin, the signal transduction associated with reactions that occur within the assembly can be extremely efficient. By controlling solution pH, it is possible to incorporate proteins into the assembly without losing the protein functionality.

Since their discovery, carbon nanotubes (CNTs) have demonstrated remarkable chemical and physical properties. CNTs are highly conductive, and they have favorable electrocatalytic properties. When confined to electrode surfaces, CNTs lower the overpotential for many kinetically unfavorable electrode reactions. Many have taken advantage of this property in the construction of different biosensors. The following will provide an overview of developments in these areas as related to glucose sensor construction and applications.

## 2. Electrostatic Assembly

The deposition of molecular layers using through-space electrostatic interactions has been an active area of research [79,80]. This technique forms uniform thin films quickly and easily from solution. Decher first described these “fuzzy nanoassemblies” in 1991, and, since then, these films have been explored for a variety of uses, including making thin films with biological activity [80–85]. The electrostatic assembly method is accomplished simply by alternately exposing a solid support that has a charged surface to solutions containing oppositely charged polyions for a short period of time. The amount of adsorbed material is self-limiting since repulsion among the similarly charged polyions will limit the amount of the polyion that will bind to the charged substrate [79,80]. The quantity of polyion adsorbed will have more than the stoichiometric number of charges relative to the substrate, resulting in the charge of the surface assembly being reversed from its initial condition as a result of adsorption of the polyion [79,86]. Once the first polyion layer has been deposited, the substrate may then be dipped into another solution containing a polyion of opposite charge. This results in adsorption of the polyion of opposite charge and another reversal of the surface charge. One deposition cycle results in the adsorption of one polycationic layer and one polyanionic layer, forming a complete bilayer [87,88]. Additional cycles of exposing the charged surface to solutions of polycations and polyanions will result in stepwise growth of the interfacial film.

The greatest advantage of the electrostatic assembly process is that, in principle, any polyion can be incorporated into the film [79,80]. The polycations and polyanions most often used are aqueous polymers, although some work has been done with organic soluble polymers, and some research has taken advantage of other types of through-space interactions (e.g., hydrogen bonding) as the driving force for adsorption [79,80]. The most commonly studied polyions are commercially available synthetic polyelectrolytes such as poly(allylamine), poly(styrenesulfonate), poly(ethyleneimine) and poly(vinyl sulfate) [79,89–92]. Proteins and/or enzymes can also be incorporated into the films readily as these biological species are also polyions, whose charge depends on the protein pI and the solution pH. Since enzymes can have an overall charge, immobilization of the enzyme into a layer is simple and requires no complex chemical reaction or derivatization scheme. In principle, electrostatic deposition of the enzyme will not result in loss of enzyme activity [79]. In addition, the enzyme may reside in close proximity to any mediators in the underlying film, making oxidation of the enzyme by mediated electron transport within the thin film possible.

Several research groups show that biopolymers can be used in the sequential development of multilayer films by electrostatic assembly. Lvov *et al.* create complex multilayer films with synthetic polyions that also incorporate biopolymers, including GluOx [93]. In this application, a complex assembly of poly-l-lysine, poly(ethylenimine), polystyrene, and glucose oxidase was created. They show that up to 25 molecular layers can be deposited by this simple electrostatic deposition. They also find that the complex assembly method is important for the successful deposition of the enzyme within the thin film, as the direct assembly of the proteins (e.g., GluOx) in the absence of the multilayer structure was difficult to achieve. Lvov *et al.* suggest that this is due to the 3-dimensional structure of the proteins, as compared to the linear structure of the synthetic polyions making the electrostatic assembly ineffective [93]. Although they don’t report applications of these multilayer assemblies, they propose that these assemblies may be used for sequential enzyme reactions, or for the vectorial transfer of electrons along the layers of the assembly.

Mizutani *et al.* coadsorb GluOx and poly-l-lysine onto an Au electrode that was first modified by the self-assembly of 3-mercaptopropanoic acid [94]. Although not a layer-by-layer assembly, they rely on electrostatics between the negatively charged interfacial monolayer and the cationic poly-l-lysine to drive the assembly process. The authors propose that the GluOx is entrapped in the poly-l-lysine matrix that is held by electrostatics to the carboxylate terminated monolayer. Using this molecular assembly, the authors are able to detect H_2_O_2_ production by oxidation at the gold substrate within one second of the introduction of glucose. This assembly had a linear response up to glucose concentrations of 0.0015 mol/L, and a detection limit of 2 × 10^−5^ mol/L.

Hou *et al.* use the layer-by-layer electrostatic deposition to prepare an interface with alternating layers of poly(4-vinylpyridine) and GluOx [96]. The enzyme is in its native form in the assembly; and, unlike Lvov *et al.*, they don’t use any synthetic polyions to coadsorb the enzyme. The poly(4-vinylpyridine), PVP, is modified by the covalent attachment of N,N-dimethlyaminomethylferrocene. In this manner, the ferrocene side-chain of the cationic PVP layer serves as a redox mediator for the glucose oxidase in the assembly. In the absence of glucose, this assembly has a current-voltage response characteristic of a confined ferrocene group. When glucose is added to the electrolyte solution, a steady state current-voltage response is observed. This current response is characteristic of a catalytic enzyme reaction. The current response of this electrode assembly scales linearly with the number of PVP-GluOx bilayers (up to 5 bilayers), with a linear response up to glucose concentrations of 0.010 mol/L and a detection limit of 1 × 10^−5^ mol/L.

Calvo *et al.* also report a layer-by-layer assembly between poly(allylamine) and GluOx [19]. In this application, the poly(allylamine) is covalently modified by attachment of ferrocene carboxylic acid, and the ferrocene side-chain acted as a redox mediator for the GluOx during the enzyme reaction. Calvo *et al.* found the current response of this modified electrode increases linearly for up to four PAA/GluOx bilayers. They also analyzed the response using Bartlett’s treatment for diffusion and reaction within a thin film [96,97]. From this analysis, they find that only a portion of the confined GluOx are accessible to the confined ferrocene mediator.

**Figure 2.**
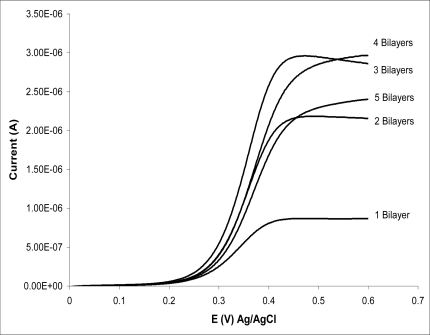
Electrode response to multiply bilayers of poly(allylammonium) chloride modified with ferrocenecarboxylic acid/Glucose Oxidase in a linear sweep voltammetry experiment. The steady state current generated at the electrode assembly increases with up to 3 bilayers. With more than 3 bilayers, the current is found to decrease with each subsequent bilayer assembly [27]. (Alice C. Harper and Mark R. Anderson, “Electrostatic Assembly of a Redox Catalysis System for Detection of Glutamate”, Electroanalysis, 2006. Copyright Wiley-VCH Verlag GmbH & Co. KgaA. Reproduced with permission).

This result is consistent with the report of Harper and Anderson, who find that the electrochemical response of multilayers of PAA (modified with ferrocene) and GluOx saturates at 3 bilayers; and, with 5 or more bilayers, the response to glucose actually decreases (Figure 2) [27].

Hou *et al*. also report layer-by-layer assembly of GluOx with PVP that has been covalently modified with an osmium bipyridine complex [98]. As with the PVP modified by ferrocene, the osmium complex serves as a redox mediator for the enzyme reaction. They find a linear response for up to 0.010 mol/L glucose concentrations, and a detection limit of 5 × 10^−5^ mol/L. By evaluating the response to various amounts of glucose, Hou found an apparent Michaelis-Menton rate constant for the enzyme reaction of 8.5 mM with 1 bilayer, and 13.5 mM for 5 bilayers. These Michaelis-Menton rate constants are consistent with values found with electrodes modified with GluOx by other methods [99]. The increase in the Michaelis-Menton rate constant with increasing numbers of bilayers is consistent with their observation that the response increases with increasing number of bilayers. Hou *et al*. suggest that the performance of these electrodes as sensors could be increased simply by increasing the number of PVP-GluOx bilayers.

This suggestion is in contrast with the results of Harper and Anderson who find that the response of the layer-by-layer assembly diminishes with more than three polycation/GluOx bilayers [27]. Harper and Anderson propose that this is the result of the enzyme reaction occurring further from the electrode surface, and this results in a decreased efficiency of coupling the electrode reaction with the enzyme reaction. Anderson also finds that the charge-transfer impedance of electrostatically assembled bilayers increases with increasing numbers of bilayers, and suggests that this is characteristic of the decreased efficiency of molecules in the adjacent solution diffusing into the interfacial assembly [100].

Zhao *et al*. use the layer-by-layer assembly method to create assemblies with alternating layers of GluOx and Prussian blue nanoparticles [101]. Prussian blue is known to catalyze the reduction of H_2_O_2_; and, by incorporating Prussian blue nanoparticles into the assembly, the overpotential for H_2_O_2_ detection is reduced. In this work, the Prussian blue nanoparticles are encased in a poly(dimethyldiallylammonium) chloride polymer to give the nanoparticles a net positive surface charge. This allows the nanoparticles to be assembled onto the electrode surface by electrostatic layer-by-layer methods. Since the overpotential for H_2_O_2_ reduction is decreased, glucose is detected more efficiently in the presence of typical interfering species.

Hooper and Anderson also use layer-by-layer electrostatic assembly as a mechanism for discriminating against interfering species [102]. In this case, they deposit poly (dimethyldiallyammonium) chloride and a poly(styrene sulfonic acid)/GluOx mixture onto the interior walls of a fused-silica capillary. Like Lvov, Hooper and Anderson find that deposition of GluOx by itself is not as efficient as depositing a polyanion/GluOx mixture. As glucose flows along the capillary, it reacts with the confined enzyme, and the H_2_O_2_ that is produced is swept down the capillary and is detected electrochemically at the capillary exit. Determination of glucose with the capillary enzyme reactor has a linear response to 0.010 mol/L glucose concentrations, and a detection limit of 5 × 10^−4^ mol/L when using a 50 μm inner-diameter capillary. By coupling this enzyme reactor capillary with a capillary electrophoresis separation, the H_2_O_2_ produced during the enzyme reaction is efficiently separated from the possible interferences present in the sample mixture.

**Figure 3.**
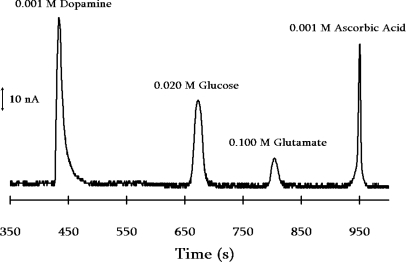
Electrophorogram obtained with a 50 μm internal diameter fused silica capillary whose interior walls have been modified by electrostatic deposition of poly(dimethyldiallyammonium)chloride and poly(styrene sulfonic acid)/enzyme mixture. For this separation, the enzyme is a mixture of glucose oxidase and glutamate oxidase. The glucose and glutamate are separated by the electrophoresis from dopamine and ascorbic acid, and the glucose and glutamate are detected by oxidation of the H_2_O_2_ generated by the enzyme reaction that occurs along the walls of the capillary [103].

As described by Willner when the enzymes are entrapped by electrodeposited polymers [25], multiple enzymes can be incorporated into the electrostatic assembly on the capillary walls to determine multiple analytes. Hooper and Anderson confine both glucose oxidase and glutamate oxidase in the electrostatic assembly on the inner walls of a capillary, allowing for the simultaneous determination of both glucose and glutamate. When conducted during a capillary electrophoresis separation, the glucose and the glutamate can be determined simultaneously and in the presence of dopamine and ascorbic acid, two common interfering species in biological samples (Figure 3).

## 3. Incorporating Carbon Nanotubes

As previously discussed with traditional electrode materials, direct heterogeneous electron transfer to glucose oxidase is kinetically slow. This is the reason for using electron transfer mediators or indirect detection for monitoring glucose with electrochemical sensors. It has been found, however, that electrodes modified with single or multi-walled carbon nanotubes (CNTs) demonstrate fast heterogeneous electron transfer kinetics as compared to that found for traditional electrodes. Incorporating CNTs into the assembly of electrochemical glucose sensors, therefore, affords the possibility of facile direct electron transfer to glucose oxidase, and a more direct determination of glucose.

There are many methods for modifying interfaces with CNTs. All of these methods aim to take advantage of the high conductivity of these materials. CNTs are known to have electrocatalytic properties and fast electron transfer rates for many redox processes. This is due to the chemical and physical properties of these materials. The structure of carbon nanotubes is unique; being similar to graphite sheets along the cylinder with properties resembling those of the basal plane of highly ordered pyrolytic graphite (HOPG). The open ends of CNTs are similar in structure and properties to the edge plane of HOPG. McCreery *et al.* studied extensively the electrochemical behavior of HOPG electrodes, and find that the edge plane has much better electron transfer properties than the basal plan [104–107]. By analogy, given the much larger proportion of edge to plane with CNTs, it is not surprising that CTNs demonstrate favorable electron transfer kinetics.

Efforts to incorporate CNTs into electrode materials include the use of a traditional carbon paste electrode design. In this case, the carbon paste is prepared using carbon nanotubes to replace the graphite when creating the paste matrix. The advantages of carbon nanotube paste electrodes (CNTPE) is the simplicity of construction, and the observation that CNTPEs maintain the favorable properties of traditional carbon paste electrodes (e.g., low background current, easy preparation and electrode renewal, and the ability to incorporate other materials into the composite matrix). Despite the simple construction, the CNTPE has enhanced electron transfer kinetics for the oxidation of several biological molecules (e.g., dopamine, dopac, and ascorbic acid) when compared to the electrochemical behavior found with a carbon paste electrode prepared with graphite. The improvement in the kinetics is observed even when only 10% of the carbon material in the composite is the multi-walled CNTs (the remaining 90% of the carbon material is graphite) [108]. The greatest improvement, however, is observed if 100% of the carbon material in the composite paste is CNTs.

Rubianes *et al.* demonstrate that the oxidation of H_2_O_2_ is also enhanced at the CNTPE compared to the H_2_O_2_ oxidation at a carbon paste electrodes that do not contain CNTs [108]. The enhanced electrochemical activity approaches that of a metalized carbon electrode [108]. Given this response to H_2_O_2_, a glucose sensor was prepared where glucose oxidase was also incorporated into the composite matrix. Production of H_2_O_2_ by the enzyme reaction in the presence of glucose generates an amperometric response proportional to the amount of glucose using this design. The response of this electrode was linear to 0.025 mol/L concentrations of glucose with a detection limit of 6.0 × 10^−4^ mol/L.

Nossol and Zarbin report creating a CNTPE electrode modified with Prussian blue. They use this electrode structure for H_2_O_2_ detection [109]. H_2_O_2_ oxidation occurs at a very low overpotential and at a high catalytic rate in the presence of Prussian blue, even when using a traditional carbon electrode. Using traditional electrode materials, however, the Prussian blue films tend to be electrochemically unstable. By creating the Prussian blue film on the CNTPE by electrochemical synthesis, a stable film formed. This is likely due to the high efficiency CNTs demonstrate for adsorption of materials to the CNT surface. This film also showed enhanced electrochemical behavior toward H_2_O_2_ oxidation. This electrode response was linear to H_2_O_2_ concentrations up to 5.0 × 10^−5^ mol/L with a detection limit of 3.27 × 10^−8^ mol/L. Using this CNTPE construction that incorporates Prussian blue, H_2_O_2_ oxidation occurred at the low overpotential of 0.0 V *vs*. Ag/AgCl. This should be compared to the oxidation overpotential for H_2_O_2_ of ∼1.0 V *vs*. Ag/AgCl found with carbon paste electrodes prepared from graphite [110].

Zhang and coworkers also create a CNT composite electrode with adsorbed Prussian blue [111,112]. In this case, the Prussian blue is created *in situ* in the presence of a dispersion of single walled CNTs. Due to the low solubility of Prussian blue, cubic crystals of Prussian blue deposit onto the single walled CNTs. The Prussian blue modified CNTs are then isolated and deposited onto a glassy carbon electrode from a chitosan electrolyte solution containing a dispersion of the modified CNTs with −1.5 V *vs*. SCE applied to the glassy carbon electrode. This electrode had a linear response to H_2_O_2_ up to concentrations of 0.0275 mol/L and a detection limit of 1.0 × 10^−8^ mol/L. A glucose sensor was similarly constructed by the addition of glucose oxidase to the chitosan dispersion that contains the Prussian blue modified CNTs prior to deposition. The response to glucose concentrations was linear to 0.0135 mol/L with a detection limit of 5.0 × 10^−6^ mol/L. The response of this electrode to glucose was constant for 4 days with this electrode, but the amperometric response to glucose degraded to ∼82% of its initial response within 10 days.

This electrode preparation is an example of another simple preparation in which the CNTs are cast onto an electrode substrate (generally a glassy carbon electrode) from a solution in which the CNTs are dispersed [111]. Due to the low solubility of CNTs in most solvents (but especially typical electrolyte solutions used for electrochemical measurements), carbon nanotubes can be applied to electrode substrates by casting from a dispersion followed by allowing the solvent to evaporate to form CNT modified electrodes that are stable to electrochemical conditions. Several research groups use this simple solvent casting method for depositing CNTs onto electrode, and casting CNTs onto electrode substrates has been shown to be an effective procedure for modifying electrodes [113–116]. Wang *et al.*, demonstrate that CNTs are very soluble in Nafion solutions and casting from Nafion solutions provides a method for coating electrodes where the amount of the CNTs can easily be varied [117]. Nafion films are also frequently used in the construction of biosensors because the charge of the Nafion polyelectrolyte film offers some ability to discriminate between species in a sample. In Wang’s application, glassy carbon electrodes modified with CNTs were prepared by casting 20 μL of a 0.5 wt.% Nafion solution containing 2 mg/mL multiwalled CNTs, and allowing the electrode to dry at room temperature for 2 hours. Even with the CNTs in the Nafion polymer, the electrode showed good electrocatalytic behavior toward H_2_O_2_ with a low applied potential of 0.0 V *vs*. Ag/AgCl. To create a glucose sensor, this electrode (after coating with CNTs from a Nafion solution) was dipped into a solution containing glucose oxidase and gluteraldehyde to create a second layer, and then allowed to dry. With this electrode preparation, an electrochemical response to glucose was observed with an applied potential of −0.05 V *vs*. Ag/AgCl. This response to glucose was good, and the electrode had good discrimination against acetaminophen, ascorbic acid and uric acid.

With the carbon nanotube paste electrodes (CNTPE), the CNTs are mixed with a binding agent to create a composite that is used to fill a reservoir of the electrode body. Using this preparation, the CNTs have a random arrangement within the matrix and there is no control over the edge *vs*. wall exposure. Similarly, when prepared by casting dispersions of CNTs onto an electrode substrate, a random arrangement of the carbon nanotubes on the surface is created. Esplandiu *et al*. study electrode materials made from composites of carbon nanotubes and epoxies [118]. Unlike the paste electrode preparation or the cast electrode preparation, the composites prepared by Esplandiu *et al.* are rigid and the orientation of the CNTs is fixed once the matrix is prepared [118]. These rigid composites are also different from carbon paste electrode construction in that the surface can be renewed (by polishing); and, once polished, the wall to edge proportions of the CNTs exposed to the solution is fixed. With this construction, the authors examine the importance that the edge *vs*. the wall of the CNT play in establishing the electrochemical behavior [118]. They find that by varying the diameter of the multiwalled CNT used when preparing the composite and while keeping the CNT length and the relative composition of the composite constant, the percentage of the wall *vs*. the edge of the CNT that is exposed at the electrode surface can be varied and controlled. From their study, a high percentage of CNT edge at the electrode surface is important to observing the enhanced electrocatalytic behavior associated with CNT modified electrodes. Further, single walled CNTs, with smaller diameter and a higher edge to wall ratio than the multi-walled CNTs, had the best electrochemical properties.

The results of Esplandiu *et al.* [119] are consistent with the findings of McCreery *et al.* [104–107]. from their work using traditional HOPG electrodes, and suggests that the best electrochemical behavior with carbon nanotube modified electrodes will be obtained if the CNTs are oriented so that the edges are preferentially exposed to the electrolyte solution.

Several research groups discuss ways of growing CNT oriented films [119–121]. For example Tu *et al.* created nanoelectrode arrays with the aligned carbon nanotubes that demonstrate excellent electrocatalytic properties [121]. Once grown, the aligned CNTs were encased in epoxy and the surface polished to expose the ends of the nanotubes so that they are flush with each other and with the encasing epoxy matrix. In this manner, they create an electrode array with millions of nanoelectrodes. By creating a low-density of carbon nanotubes when the nanoelectrode array is formed, the diffusion layers associated with each individual nanoelectrode will not overlap, and the nanoelectrode array demonstrates the steady-state current response of an individual nanoelectrode. Since there are millions of these nanoelectrodes in the array, the total current measured is on the order of hundreds of nanoamperes and is easily measured. By exposing just the end of the carbon nanotubes, an array electrode with good electrocatalytic properties is created.

Tu *et al.* were able to use this array electrode to construct a glucose sensor using this preparation method followed by attaching glucose oxidase to the “broken” ends of the carbon nanotubes [120]. This is accomplished by electrochemically treating the CNT nanoelectrode array to create carboxyl groups at the exposed ends, and then subsequently reacting the CNT nanoelectrode array with glucose oxidase in the presence of 1-ethyl-3-(3-dimethylaminopropyl) carbodiimide and N-hydroxy-sulf-succinimide to couple the glucose oxidase through amide linkages to the carboxyl groups at the exposed end of the carbon nanotubes. The glucose sensor that was produced was shown to have a relatively fast response that is linear to 0.030 mol/L, and a limit of detection of 8.0 × 10^−5^ mol/L. Because of the electrocatalytic effect of the CNTs, this sensor can operate at relatively low applied potentials for the oxidation of the H_2_O_2_ that is generated by the enzyme reaction (−0.2 V *vs*. Ag/AgCl). The low applied potential for the H_2_O_2_ detection provides good discrimination against common interferences—ascorbic acid, uric acid and acetaminophen. This is an example of covalently modifying the CNT. In this case, the sensor is still detecting the glucose indirectly by measuring the oxidation of H_2_O_2_, but the electrocatalytic effect of the CNTs allows the measurement to be made at low overpotential.

Chattapadhyay *et al.* report another method for creating electrodes modified with aligned CNTs that extend away from the substrate surface [122,123]. They refer to their preparation as single wall nanotube (SWNT) forests, indicating a relatively high density of carbon nanotubes along the substrate. Briefly, the SWNT are assembled on a pyrolitic graphite substrates that have previously been coated with a layer prepared from Nafion and precipitated Fe(OH)_3_. The deposited CNTs are found to orient with the long axis of the nanotube perpendicular to the substrate. In this arrangement, as Tu *et al.* demonstrated [120], standard carbodiimide coupling chemistry can be used to attach biopolymers (e.g., glucose oxidase, myoglobin, horseradish peroxidase, *etc*.) to the terminal carboxyl group on the carbon nanotubes through an amide. As with Tu *et al.*, glucose is determined with these CNT forest electrodes by monitoring the production of H_2_O_2_ on exposure to glucose.

Alternatively, Gooding *et al.* modify a gold substrate using the self-assembly of cysteamine to create a homogeneous interface with exposed amine groups [124]. Again, using carbodiimide coupling chemistry, single-wall CNTs that have been modified by sonication in acid at elevated temperatures to create carboxyl functionality at the ends can be attached to the amine groups at the interface. This procedure, like that of Chattapadhyay *et al.* creates an interface with a high density of aligned CNTs with the CNTs extending vertically away from the substrate. Using this method, Gooding *et al.* create single-walled carbon nanotube forests to which glucose oxidase is attached. They produce two types of electrodes by this procedure. Like Chattapdhyay, Gooding attaches the native GluOx to the activated ends of the vertically aligned carbon nanotubes. With this preparation, they observe the direct electron transfer to the FAD redox center of the GluOx. They propose that this is due to the proximity of the FAD site to the end of the CNT and to the favorable electrocatalytic properties of the CNT being able to overcome the distance between the carbon nanotube and the FAD redox center of the enzyme. They find that this electrode construction has a direct electron transfer reaction to the GluOx that occurs with a 0.3 s^−1^ heterogeneous rate constant. Alternatively, Gooding attaches denatured GluOx to the activated end of the CNT by carbodiimide coupling; and, then, they reconstitute the three-dimensional structure of the GluOx after it is attached to the CNT. Again, they observe direct electron transfer from the electrode to the GluOx; however, with this preparation, Gooding *et al.* report a much higher heterogeneous rate constant of 9 s^−1^ for the electrode reaction. In this case, the CNT is thought to penetrate the GluOx structure so that it is very close to the active FAD site of the enzyme. The proximity of the nanotube end to the FAD site within the enzyme facilitates the favorable electron transfer kinetics observed.

Wallace *et al.* create aligned carbon nanotubes by pyrolyzing iron(II)-phthalocyanine under an Ar/H_2_ atmosphere at 900 °C [125]. In this preparation, Fe particles that result from the pyrolysis serve as the catalyst around which the nanotube grows. The Fe particle remains at the tip of the CNT after growth is terminated, and the authors suggest that the Fe particle is partly responsible for the low overpotential observed with these electrode structures for the oxidation of H_2_O_2_. This was tested by removing the Fe particle (by acid treatment at elevated temperatures). Following removal of the Fe, the electrochemical response to H_2_O_2_ is completely gone. This result suggests that the Fe particle found at the tip of the nanotube is responsible for the electrocatalytic behavior of this electrode toward H_2_O_2_.

Both Wallace *et al.* and Tu *et al.* take advantage of metallic nanoparticles to initiate growth of carbon nanotubes at an electrode surface [119–121,125]. Wallace *et al.* also suggest that the presence of the metallic nanoparticle is at least partially responsible for the observed electrochemical behavior of these modified electrodes. Others also use CNTs modified with metallic nanoparticles in attempts to combine the electrocatalytic properties of the CNT and that of the metallic nanoparticles. For example, Wang *et al.* show that H_2_O_2_ can be oxidized at glassy carbon electrodes modified with CNTs despite the fact that H_2_O_2_ is not normally oxidizable at glassy carbon [117]. H_2_O_2_ oxidation is frequently measured with Pt electrodes as the overpotential for H_2_O_2_ oxidation is lower at Pt (∼0.4 V *vs*. Ag/AgCl) [103] than at bulk carbon electrodes (∼1.0 V *vs*. Ag/AgCl). Luong *et al.* modify a glassy carbon electrode with both CNTs and Pt nanoparticles in an effort to leverage the electrocatalytic properties of both these materials in the oxidation of H_2_O_2_ [127]. Here, glassy carbon electrodes are modified by casting a solution that contains Pt nanoparticles, CNTs, and a low concentration of Nafion onto the electrode surface. After drying, they find that this structure has a very high electrocatalytic effect toward the oxidation of H_2_O_2_, having a detection limit of 2.5 × 10^−8^ mol/L and a linear range up to 2.0 × 10^−3^ mol/L. When an overcoat of glucose oxidase and gluteraldehyde is applied to this electrode, a linear response to glucose concentrations of up to 5.0 × 10^−3^ mol/L and a detection limit of 5.0 × 10^−7^ mol/L are found.

Fang *et al.* also create modified electrodes that incorporate CNTs and Pt nanoparticles [127]. They grow electrodes having aligned CNTs using a procedure similar to that described by Wallace [125]. Once prepared, the aligned CNT modified glassy carbon was dipped into an electrolyte solutions containing H_2_PtCl_6_ and NaNO_3_, and the potential ramped from 0.0 to 0.6 V *vs*. Ag/AgCl to deposit Pt nanoparticles onto the CNTs. The aligned CNT/Pt nanoparticle electrode was then dipped into a solution containing chitosan, glucose oxidase, and CdS colloids. While exposed to this solution, the electrochemical reduction of H^+^ lowered the pH local to the electrodes surface causing the chitosan to precipitate and trap the glucose oxidase and CdS at the electrode surface. With this structure, Fang *et al.* observe the direct electron transfer to the FAD/FADH_2_ redox couple of the enzyme [127]. The authors state that the CdS nanoparticles facilitate the direct electron transport from the highly conductive nanotubes to the enzyme, and the electrocatalytic properties of the Pt nanoparticles also facilitate the heterogeneous reaction. The catalytic behavior of the enzyme is not affected by the assembly structure, and the electrode showed no obvious response from typical interfering compounds. The authors suggest that this structure can be used for reagentless biosensing.

Direct electron transport to the redox center of an enzyme offers the opportunity for creating biosensors with superior time responses. Gooding *et al.* demonstrate that by reconstituting the enzyme’s three-dimensional structure after attaching it to a CNT results in superior heterogeneous kinetics for the direct electron transfer to the FAD redox center of glucose oxidase [124]. Fang use both CNTs and CdS nanoparticles to facilitate the direct electron transport to the enzyme [127]. Guiseppi-Elie *et al.* also report the direct electron transfer from CNT modified electrodes to glucose oxidase [128]. They find that both FAD and glucose oxidase adsorb to single walled carbon nanotubes that are cast onto glassy carbon electrodes. Once prepared, these single walled CNT modified electrodes had current responses characteristic of the redox chemistry of FAD/FADH_2_. When the glucose oxidase modified structure is exposed to glucose in an oxygen saturated solution, a current response consistent with molecular oxygen being consumed at the interface by the enzymatic conversion of glucose to gluconolactone is observed. The authors speculate that the direct electron transfer to glucose oxidase is a result of the fibrils of the cast CNT film coming within tunneling distance of the FAD portion of the glucose oxidase enzyme. Like Gooding, Guiseppi-Elie *et al.* proposed that the single-walled CNTs penetrate the native structure of the enzyme to facilitate the direct electron transfer. Unlike Gooding, Guiseppi-Elie propose that this happens without having to denature and then reconstitute the enzyme structure around the carbon nanotube.

Several research groups report using the simplicity of the electrostatic assembly method for creating a carbon nanotube assembly on an electrode surface [129–131]. In each case, the walls of the carbon nanotube are first modified to establish charge so that the CNTs can subsequently be deposited onto the substrate by electrostatic interactions. As demonstrated by others, many materials will adsorb strongly to CNT walls. Different methods have been reported for establishing the charge along the CNT wall. Dong *et al.* treat CNTs by sonication in concentrated HNO_3_ and H_2_SO_4_ [129]. Similarly, Guo *et al.* reflux CNTs in concentrated HNO_3_ for 5 hours to prepare them for the electrostatic assembly [131]. These treatments both shorten the CNTs and create carboxylic acid groups along the walls. When exposed to basic solutions, the carboxylic acid groups deprotonate and this establishes a negatively charged surface for electrostatic assembly [129]. Others take advantage of the surface properties of the CNTs to simply adsorb polyelectrolytes to the surface [130]. Tsai *et al.* comment that noncovalent adsorption of polyelectrolytes to CNTs does not alter the electronic structure of the CNTs where as covalent coupling might [130].

Although they did not construct a glucose sensor, the method used by Guo *et al.* is typical of these electrostatic CNT assemblies [131]. They initially treat multi-walled CNTs by refluxing in concentrated HNO_3_ solution to create carboxylic acid groups along the walls of the CNTs. These negatively charged nanotubes were then cast onto a gold electrode surface and the solvent allowed to evaporate, leaving behind a negatively charged CNT modified substrate. The CNT modified electrode surface was then exposed to a solution of poly(diallyldimethyl ammonium chloride) for 1 hour so that the cationic PDDA could electrostatically assemble onto the negatively charge CNTs. This assembly was then immersed in a buffered solution containing cholesterol oxidase. At the pH of the buffer, the cholesterol oxidase enzyme is negatively charged and assembles onto the cationic PDDA. Like a glucose oxidase based sensor, this measurement monitors the oxidation of the H_2_O_2_ produced in the enzyme reaction. They find that this assembly is more sensitive than other cholesterol biosensors with a lower Michaelis-Menton constant [131].

Deng *et al.* construct a glucose electrochemical sensor by first adsorbing poly(allylamine) ferrocene (Fc-PAH) onto the walls of CNTs that had been previously functionalized with carboxylic acid groups by sonication in concentrated HNO_3_ [129]. By adsorbing the Fc-PAH to the negatively charge CNTs, the authors create a net positive charge on the polymer coated nanotubes walls, and they also introduced a ferrocene redox catalyst into the assembly. The Fc-PAH modified CNTs were then electrostatically deposited onto an Indium Tin Oxide (ITO) substrate. This modified ITO was subsequently modified by electrostatic deposition of negatively charged glucose oxidase. By alternately exposing the modified electrode to solutions of Fc-PAH modified CNTs and solutions of glucose oxidase, multiple bilayers of these 2 materials were deposited onto the ITO substrate. The sensitivity of these assemblies scaled with the number of bilayers deposited, and resulted in a 6–10 fold increase in the measured catalytic current for the oxidation of glucose.

Tsai *et al.* take advantage of the high surface area of CNTs to adsorb glucose oxidase to the nanotube [130]. This is done using the sodium cholate suspension dialysis method [132,133]. This method results in adsorption of the enzyme onto the CNTs without the need to alter the structure of the CNT while retaining the biological activity of the enzyme. The glucose oxidase modified CNTs were deposited onto gold electrodes by the electrostatic layer-by-layer method with alternating layers of the cationic redox polymer poly [vinylpyridine)Os(bipyridyl)_2_Cl]. The redox polymer in this case provides the cationic portion of the electrostatically held bilayer, and it also serves as a redox mediator for the catalytic reaction of glucose with the enzyme. With these assemblies, current densities that are nearly twice that found in the absence of the CNTs were observed. A six bilayer film of this construction is reported to have a sensitivity of 56 μA/mM-cm^2^ at 0.005 mol/L glucose. Tsai *et al.* report that this response is “among the highest ever reported” for layer-by-layer glucose sensors [130]. They also suggest that the sensitivity can be controlled by changing the number of bilayers deposited onto the electrode substrate. This is in contrast to others who find that the response of layer-by-layer assemblies that incorporate enzymes peaks at a finite number of bilayers and decreases with additional bilayers [27]. This difference suggests that the presence of the carbon nanotubes improves the electrical connection between the substrate electrode and the bilayer assemblies, providing more efficient electron diffusion through the assembly structure [130].

## 4. Conclusion

Glucose sensors based on electrochemical signal transduction have been an area of active study since the first publication in 1962. Despite its maturity, research in the area of electrochemical glucose sensors continues to thrive, and new innovations appear regularly. These innovations, however, are generally directed at solving some of the fundamental difficulties observed over the years with glucose sensors—e.g., overcoming the kinetic limitations associated with direct electron transfer to the glucose oxidase enzyme, lowering the overpotential for H_2_O_2_ oxidation, or creating interfacial molecular architectures to optimize the signal transduction from the enzyme reaction—and that is the case with innovations using electrostatic assembly for confining the enzyme and the redox mediator system to the electrode surface, and with innovations involving the use of carbon nanotubes.

In this review, we’ve considered innovations offered by a simple method for developing interfacial thin-film architectures using the electrostatic attraction of polyelectrolytes. By incorporating enzymes into the polyelectrolyte assembly, several authors have shown that enzymes and redox mediator assemblies can be confined to molecular films attached to an electrode. The proximity of the mediation system to the electrode surface offers advantages to the sensor response time; however, there is some disagreement in the literature about the value of multiple bilayers toward the efficiency of this construction. If CNTs are incorporated into the layer-by-layer assembly, the efficiency of the molecular structure is combined with the electronic properties of the CNTs. This establishes more efficient electron transfer through the layer-by-layer assembly, and possibly generates a better response. The role that the CNT plays in the electron diffusion efficiency in these layer-by-layer assemblies needs to be more thoroughly investigated. It has also been demonstrated that the electrostatic assembly can be incorporated into analytical systems other than electrodes—for example, separation capillaries. When coupled to a separation capillary, multiple enzymes can be used and detection of multiple enzyme substrates accomplished.

Incorporating carbon nanotubes into the sensor assembly offers the advantages of the improved electrocatalytic properties of these materials. This effectively lowers the oxidation overpotential for the indirect detection of glucose by H_2_O_2_ oxidation and creates conditions that are favorable for the discrimination of H_2_O_2_ from common interfering species. The small size of carbon nanotubes offers the potential that these materials can penetrate the enzyme structure without disrupting its functional behavior, and several authors report that this attribute allows for the direct electron transport to the FAD active site of the glucose oxidase enzyme. It is not clear from these studies, however, if CNT penetration into the enzyme structure is required to observe the enhanced electrocatalytic behavior. Similarly, it is not clear from the literature reports if the size of the CNT allows a close proximity of the CNT to the redox active FAD center of the enzyme, or if the CNT creates conditions favorable for longer tunneling distances that leads to the electrocatalytic behavior.

Both of the introduction of carbon nanotubes and the electrode preparation by layer-by-layer electrostatic assembly are areas of research that address problems that have limited the development of electrochemical glucose sensors for many years. Although not exhaustive, this review touches on the principle contributions to the creation of glucose sensors that electrostatic assembly and carbon nanotubes have made to date, and provides insight into how these areas may continue to add to the development of electrochemical glucose sensors.

## Figures and Tables

**Scheme 1.**
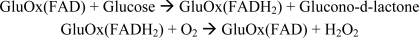


**Table 1.** Examples of redox mediators used with electrochemical sensors that incorporate Glucose Oxidase.

**Mediator**	**E^o^*vs*. Ag/AgCl**
Benzyl viologen [45]	−0.370 V
Indigo disulfonate [45]	−0.188 V
Methylene blue [46]	0.217 V
2,5-dihydroxybenzoquinone [46]	0.137 V
Ferrocenecarboxaldehyde [25,26]	0.518 V
Ferrocenemethanol [25,26]	0.216 V
